# Utilizing mitochondrial genome sequences to understand population diversity among *Trichoderma* species

**DOI:** 10.3389/ffunb.2026.1810157

**Published:** 2026-05-15

**Authors:** Christopher M. Wallis, Jianchi Chen, Nalong Mekdara

**Affiliations:** Crop Diseases, Pests and Genetics Research Unit, San Joaquin Valley Agricultural Sciences Center, U.S. Department of Agriculture- Agricultural Research Service, Parlier, CA, United States

**Keywords:** biological control agents, bot canker, dieback, fungal trunk disease, grapevine

## Abstract

Biopesticides, including those containing various *Trichoderma* species, may provide improved control of fungal diseases of woody perennials including grapevines. Thus, *Trichoderma* isolates were obtained from grapevines in California, U.S.A., with eight strains demonstrated to provide biological control against fungal pathogens. These were determined via phylogenetic analyses to be one of six different species: *Trichoderma asperellum*, *Trichoderma capillare*, *Trichoderma harzianum*, *Trichoderma saturnisporopsis*, and two novel *Trichoderma species* (*Trichoderma* sp. DL1–3 and *Trichoderma* sp. PAR10). However, little is known about strain genetic diversity. Therefore, efforts were undertaken to characterize and compare obtained mitochondrial genomes with those previously published from related *Trichoderma* species. For this effort, the mitochondrial genomes of *T. asperellum* strain TLI, *T. capillare* strains KC2–2 and SLO1-1, and *T. saturnisporopsis* strain RSI were obtained and annotated, and compared to the other Californian strains and 23 other *Trichoderma* species genomes. All analyzed species had the same major protein coding genes, rRNA regions, and tRNA coding sequences. Further, specific introns were observed to be associated with distinct clades within the *Trichoderma* genus, and these could be used to develop markers to quickly assign an unknown *Trichoderma* into a specific clade. Knowledge of *Trichoderma* mitochondrial genomes should improve understanding about this fungal species.

## 1 Introduction

Fungal pathogens negatively affect yields in most crop plants and may cause progressive declines in perennial woody plants such as grapevines (Baumgartner et al., 2019; Wallis et al., 2022a). In addition to chemical pesticides, biopesticides, including those that utilize beneficial endophytes of the genus *Trichoderma*, may provide control as part of an integrated pest management program (Woo et al., 2014; Verma et al., 2017). *Trichoderma* fungi perform their biocontrol functions by directly predating or actively antagonizing other fungi, including pathogenic fungi, and may also cause indirect inhibition of microbial growth via production of specialized metabolites (Harman et al., 2004; Guzman-Guzman et al., 2019).

Despite the promise of *Trichoderma* as biopesticide agents, and the use of fungal members of this clade for other industrial purposes such as bioremediation and biofuel production, more information about their genomics is needed to understand the natural diversity in the genus (Woo et al., 2014; Verman et al., 2017). Towards this end, examinations of the mitochondrial genomes would be a good starting point, as these are ideal to analyze due to their relatively small sizes, high copy numbers, irreversible gene loss, limited recombination events, and high mutations rates that lead to accelerated evolution compared to nuclear genomes (Burger et al., 2003). In other words, until further improvements in the sequencing and annotation of nuclear genomes comes of age, mitochondrial genome sequencing could provide useful information about individual species and strain diversity.

Fungal mitochondrial genomes contain 14 conserved core genes involved in functional proteins involved in oxidative phosphorylation and electron transport, two genes for subunits of ribosomal RNA (the small and large subunits), and a variety of genes coding for transfer RNAs (Gray et al., 1999; Saraste, 1999). Fungal mitochondrial genomes appear generally more homogenous in size than plants or animals (Barr et al., 2005), and fungal mitochondrial genomes exhibit recombination signals similar to that of plants (Basse, 2010; Aguileta et al., 2014; Wu and Hao, 2019), albeit with introns that belong to group I versus group II (Lang et al., 2007).

In this study, the main objective was to compare mitochondrial genomes of grapevine-isolated strains of *Trichoderma* to observe evolutionary history and genetic diversity of these against similar available species with the potential of endophytically infecting plant hosts. The analyzed strains, *Trichoderma asperellum* TLI, *Trichoderma capillare* KC2–2 and SLO1-1, *Trichoderma harzianum* KC1–1 and PAR3, *Trichoderma saturnisporopsis* RSI, *Trichoderma* sp. DL1–3 and *Trichoderma* sp. PAR10. These were all demonstrated to have biological control activity when applied to grapevines both in greenhouse and field studies (Antrim et al., 2025; Wallis et al., 2025). The mitochondrial genomes for the strains KC1-1, PAR3, DL1–3 and PAR10 have been previously published (Wallis et al., 2022b; Wallis and Chen, 2025), and the remaining strains will be described in this study.

Previously, similar efforts to compare *Trichoderma* mitochondrial genomes have been undertaken. In one study, Kwak (2020) compared the mitochondrial genome of *Trichoderma atroviride* strain ATCC 26799 with four other *Trichoderma* sp. mitochondrial genomes, and determined general similarities but key differences in annotations, namely no annotated large subunit rRNA in *Trichoderma asperellum* strain B05 and no *atp9* gene annotated in *Trichoderma gamsii* KUC1747. Distinct introns also were noted among strains, with most occurring in the *cox1* gene (Kwak, 2020). In a more expanded study, Wang et al. (2024) compared 60 different *Trichoderma* mitochondrial genomes, with the majority being new isolates from China, and observed a consistent 15 core protein coding genes in each, 2 rRNAs, and 16–30 tRNAs. Numerous introns also were noted (Wang et al., 2024). Six different evolutionary branches were obtained via analyzing the mitochondrial genomes, with mutation hotspots near the regions encoding *nad2*, *cox2*, and *nad5* (Wang et al., 2024). Positive evolutionary selection appeared to be occurring with the genes *atp9*, *cob*, *nad4L*, *nad5*, and *rps3* (Wang et al., 2024).

This study will utilize a similar approach to previous efforts to examine whether the placement of the newly described mitochondrial genomes for strains TLI, KC2-2, SLO1-1, KC1–1 and PAR3 can be made. Species included in this study from the grapevine-isolates are different than those used by Kwak (2020) and Wang et al. (2024) with the exception of *T. asperellum* and *T. harzianum*, so findings should build off of these studies and further improve overall understanding of *Trichoderma* mitochondrial genomes. Results will facilitate improved understanding of *Trichoderma* evolution and could demonstrate the ability to use mitochondrial genomes in identifying newly obtained strains to *Trichoderma* clades, species, and strains.

## 2 Materials and methods

### 2.1 Fungal strains and culturing conditions

The *Trichoderma* strains DL1-3, KC1-1, KC2-2, PAR3, PAR10, RSI, SLO1–1 and TLI were collected from vineyards throughout central California in the summer of 2020 as described by Wallis et al. (2025). Cultures were maintained at 25 °C in darkness on potato dextrose agar (BD Difco, Franklin Lakes, NJ, USA) and replated every week. Morphological assessments further confirmed the cultures that belonged in the *Trichoderma* genus following a dichotomous key (Samuels and Hebbar, 2016). Further identification of these strains to sequences occurred using the methodology of Cai and Druzhinina (2021), based on sequence data obtained as outlined below and described further in Antrim et al. (2025) and Wallis et al. (2025). In brief, obtained *ITS* sequences for each strain was first confirmed to be greater than 76% with at least one known *Trichoderma* species to place these in the genus, and then obtained *rpb2* and *tef1* genes from the strains were compared with a collection of known species, with percent identities over 99% for *rpb2* and over 97% for *tef1* resulting in a positive placement of a strain within a species (Cai and Druzhinina, 2021). If not, phylogenetic trees would be examined to determine if an isolate is a putative novel species (Cai and Druzhinina, 2021).

### 2.2 DNA analysis and mitochondrial genome annotation

The strains DL1-3, KC1-1, PAR3, and PAR10 had mitochondrial genome obtained as previously described (Wallis et al., 2022b; Wallis and Chen, 2025). For KC2-2, RSI, SLO1–1 and TLI, DNA extraction was made using a NucleoSpin (McNeary-Nagel, Allentown, PA, USA) Plant II mini kit following instructions for fungal material, and a Qubit (Thermo-Fisher Scientific, Waltham, MA, USA) and a Tape Station (Agilent, Santa Clara, CA, USA) to assess DNA quality and quantity following manufacturer’s protocols. For RSI, SLO1–1 and TLI, next-generation sequencing was performed by a commercial source (Novogene, Davis, CA, USA) that used an Illumina (San Diego, CA, USA) NovoSeq 6000 instrument using manufacturer’s protocols for all strains except KC2-2. The KC2–2 sequence was obtained by a different commercial source (Plasmidsaurus Inc., Louisville, KY, USA) that used the PromethION (Oxford Nanopore, Oxford, UK) system using default manufacturer’s protocols.

For KC2-2, assembly was performed by Plasmidsaurus. In brief, the bottom 5% worst fastq reads were removed by Fitlong (ver. 0.2.1) (Wick, 2017), followed by assembly with Flye (ver. 2.9.1) (Kolmogorov et al., 2019) with parameters selected for high-quality reads and self-polishing, and lastly brief annotation by Augustus (ver. 3.5.0) (Stanke et al., 2008) and BLAST+ (ver. 2.15.0) (Camacho et al., 2009) against the UniProt database (ver. 2024_04; https://www.uniprot.org/) was performed. For Illumina sequences of RSI, SLO1–1 and TLI, the SPAdes (ver. 3.14.0) assembler was used to assembly the raw reads (Bankevich et al., 2012). A coverage map was obtained using BWA (Li and Durbin, 2009) to obtain a.sam file that was uploaded into Tablet (Milne et al., 2013) for observation of sequence reads over the contig associated with the mitochondrial genome to observe coverage across it (**Supplementary Figure 1**).

Following all assemblies, QUAST (ver. 5.2.0) and BUSCO (ver. 5.7.1) determined quality and completeness (Gurevich et al., 2013; Simao et al., 2015). A stand-alone BLAST+ (ver. 2.7.1) using the mitochondrial genome from *Trichoderma harzianum* (GenBank accession MN564945.1) as a query was used against generated contigs to identify the one containing the mitochondrial genome, which was examined to check for the presence of all major coding sequences. This contig was further verified to be circular by observing the contigs (the.fastg files from the assemblies) in Bandage (version 0.9.0) as well (Wick et al., 2015). The mitochondrial genome contigs were downloaded and screened for an overlap in sequencing, which was trimmed manually (Wallis et al., 2022b). Circularity was further confirmed by using BLAST+ against known, annotated mitochondrial genomes and each genome itself to ensure the manual trimming only removed overlapping sequences (Wallis et al., 2022b).

The online tool GeSeq (https://chlorobox.mpimp-golm.mpg.de/geseq.html) (ver. 2.03) was used to perform initial annotation of the mitochondrial genomes (Tillich et al., 2017). Protein and rRNA coding sequences were identified using the BLAT (ver. 35) search module (both BLATN and BLATX), and ARAGORN (ver. 1.2.38) or ARWEN (ver. 1.2.3) identified tRNA sequences. The RNA weasel webserver (https://megasun.bch.umontreal.ca/apps/rnaweasel/), which uses ERPIN (ver. 5.2.1) was used to further confirm tRNAs and to identify and putative characterize possible introns to type (Gautheret and Lambert, 2001; Lang et al., 2007). The Proksee Map Builder (version 1.4.4) was used to visualize the final annotated mitochondrial genomes, with options to display GC content selected (Grant et al., 2023).

### 2.3 Comparisons among *Trichoderma* mitochondrial genomes

For comparison among mitochondrial genomes of different *Trichoderma* species, the annotated genomes described above and existing, published mitochondrial genomes were obtained from NCBI GenBank (https://www.ncbi.nlm.nih.gov/genbank/), with descriptions in **Table 1**. All sequences were re-annotated according to the methods described above to ensure consistency among annotations. For each gene in each mitochondrial sequence was verified using BLAST+ (Camacho et al., 2009), and manual adjustments were made to ensure the complete gene was identified, that is, the gene had both the same start and end codons as published *Trichoderma* mitochondrial genomes had. The sequences also were repositioned to have the start of the *cox1* gene to be the origin. The order and presence of the genes was then confirmed by examining the newly generated annotation files.

**Table 1:** Fungal mitochondrial genomes analyzed in this study.

Species	Isolate	Accession number	Citation
*Trichoderma asperellum*	TLI	PV340230.1	this study
*Trichoderma capillare*	KC2-2	PV245924.1	this study
*Trichoderma capillare*	SLO1-1	PV245923.1	this study
*Trichoderma harzianum*	KC1-1	PV137761.1	Wallis et al., 2025
*Trichoderma harzianum*	PAR3	MZ713368.1	Wallis et al., 2022b
*Trichoderma saturnisporopsis*	RSI	PV340231.1	this study
*Trichoderma* sp. DL1-3	DL1-3	PV137760.1	Wallis et al., 2025
*Trichoderma* sp. PAR10	PAR10	PV137762.1	Wallis et al., 2025
*Trichoderma afroharzianum*	YN065	PP952384.1	Wang et al., 2024
*Trichoderma asperelloides*	ZJ116	PP952381.1	Wang et al., 2024
*Trichoderma asperellum*	FT101	CP084950.1	Li et al., 2021
*Trichoderma atroviride*	ATCC 26799	MN125601.1	Kwak, 2020
*Trichoderma breve*	T069	PP933710.1	Wang et al., 2024
*Trichoderma brevicompactum*	HA032	PP952393.1	Wang et al., 2024
*Trichoderma citrinoviride*	IMI 91793	OW971927.1	Buddie et al., 2025
*Trichoderma gamsii*	KUC1747	KU687109.1	Wang et al., 2024
*Trichoderma ghanense*	SC106	PP952402.1	Wang et al., 2024
*Trichoderma gracile*	HK011	PP952394.1	Wang et al., 2024
*Trichoderma hamatum*	ThamA	MF287973.1	Wang et al., 2024
*Trichoderma harzianum*	CBS 226.95	MN564945.1	Kwak, 2021
*Trichoderma koningiopsis*	POS7	MT816499.1	Castrillo et al., 2023
*Trichoderma lixii*	MUT3171	MT495248.1	Venice et al., 2020
*Trichoderma longibrachiatum*	XJ011	PP952404.1	Wang et al., 2024
*Trichoderma reesei*	QM 9414	AF447590.1	Chambergo et al., 2002
*Trichoderma simmonsii*	GH-Sj1	MZ292901.1	Chung et al., 2022
*Trichoderma velutinum*	FJ002	PP952389.1	Wang et al., 2024
*Trichoderma virens*	Gv29-8	CP071114.1	Li et al., 2021
*Fusarium globosum*	CBS 428.97	MT010913.1	Brankovics et al., 2020

Following this, the individual protein-coding genes from all the *Trichoderma* species included in this study were compiled into one fasta file (i.e. one fasta file per coding sequence) along with the mitochondrial sequence of *Fusarium globosum* as an outgroup (**Table 1**). MEGA (version 11.0.13) (Tamura et al., 2021) was then used to align the sequence for each gene at a time using the MUSCLE (ver. 3.8.425) (Edgar, 2022) alignment algorithm with default settings. This allowed the confirmation of introns in certain species, unique sequence features, and potential sequencing errors. The identified introns were subsequently removed to make a new exon only fasta file for each coding sequence for the phylogenetic analyses.

In order to visualize differences between *Trichoderma* clades, and species within them, the mitogenomes for representative strains were made using the Proksee Map Builder, with the mitochondrial genomes displayed linearly. Notations were added manually to show where differences in introns and tRNAs where present on these mitochondrial genome maps.

### 2.4 Phylogenetic analyses

MEGA (Tamura et al., 2021) then was used to align the grapevine-isolated *Trichoderma* strains with existing individual *Trichoderma* mitochondrial protein sequences (exons only, and a sequence of *Fusarium globosum* was included as an outgroup), as well as combined file with all the coding sequences, using MUSCLE (Edgar, 2022) with default settings. The MEGA file generated from this was used to perform a phylogenetic analysis to form a maximum likelihood tree, with bootstrapping including 500 replications and using the Tamura-Nei model.

Additionally, FastANI (ver. 1.34) (Jain et al., 2018) with default settings was used on the unprocessed whole genome sequences in **Supplementary Table 1** to arrange the species in a phylogenetic tree based on average nucleotide identity (ANI) for comparison. The output from FastANI, the generated pairwise distances, was imported into MEGA to visualize the phylogenetic tree using the construct neighbor-joining tree function. Furthermore, Orthofinder (version 3.1.0) (Emms and Kelly, 2015, Emms and Kelly, 2019) was used on the whole genome sequences to use discovered orthologs to form phylogenetic trees, with default settings used. Orthofinder used Diamond (ver. 2.1.24) (Buchfink et al., 2015) as the search program, FAMSA2 (ver. 2.5.2) (Deorowicz et al., 2016) as the multiple sequence alignment program, and STRIDE (ver. 1.0) (Emms and Kelly, 2017), STAG (ver. 1.0.0) (Emms and Kelly, 2019) and FastTree (ver. 2.2) (Price et al., 2010) to generate the phylogenetic tree. Figtree (ver. 1.4.4) (https://tree.bio.ed.ac.uk/software/figtree/) was used to visualize the phylogenetic tree file from Orthofinder.

## 3 Results

### 3.1 Mitochondrial genome annotations for strains KC2-2, RSI, SLO1-1, and TLI

For mitochondrial genome assembly, next generation sequencing obtained 43.6 GB of data for KC2-2, 41.8 GB for RSI, 12.3 GB of data for SLO1-1, and 24.2 GB for TLI.

**Figure 1:**
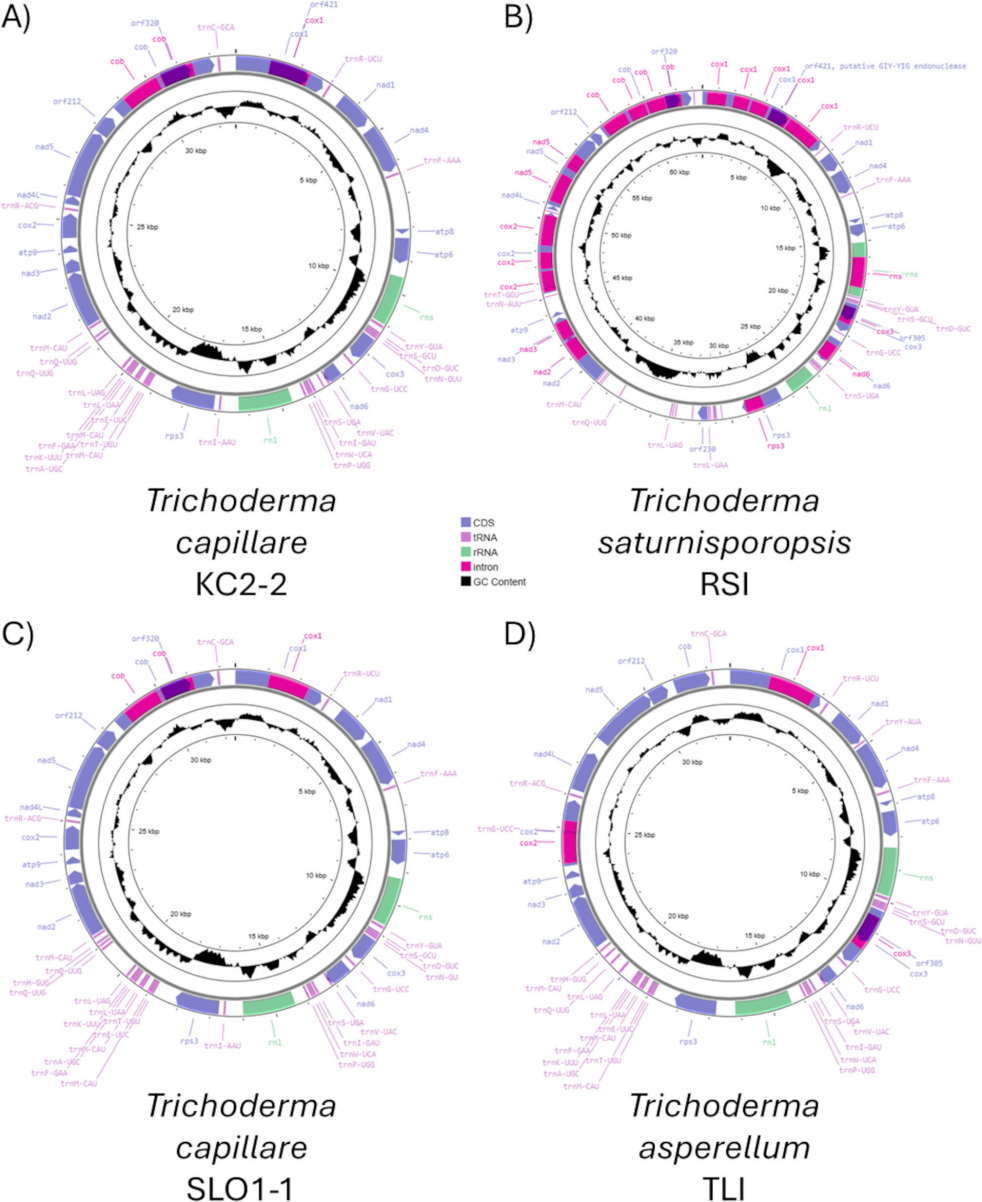
Annotation maps of the mitochondrial genomes for **(A)** *Trichoderma capillare* strain KC2-2, **(B)** *Trichoderma saturnisporopsis* strain RSI, **(C)** *Trichoderma capillare* SLO1-1, and **(D)** *Trichoderma asperellum* TLI. Coding sequences were all on the heavy strand, and the GC content is shown in the middle circle. Note for SLO1–1 not all tRNAs are labelled, but details of these are provided in **Table 2**.

Following assembly, the contig identified as being the mitochondrial genome had an average nucleotide coverage of greater than 100x for KC2-2 (the exact coverage graph was not provided by Plasmidsaurus), 798x for RSI, 1, 438x for SLO1-1, and 506x for TLI. The final completed, circular mitochondrial genomes were 32, 779 bp for KC2-2, 63, 626 bp for RSI, 32, 467 bp for SLO1-1, and 32, 716 bp for TLI (**Figure 1**). The base composition for each strain is provided on **Figure 1**, with GC% typical for Trichoderma species at 27.7% for KC2-2, 27.6% for RSI, 27.4% for SLO1-1, and 27.8% for TLI.

Mitochondrial genome annotations revealed that all four strains had three ATP synthase (*apt*) coding regions, one apocytochrome b (*cob*) coding region, three cytochrome c oxidase (*cox*) coding regions, seven NADH dehydrogenase (*nad*) coding regions, 26 transfer RNA genes, two ribosomal RNA genes, and the *rps3* gene (**Figure 1**). All coding regions were on the heavy strand. The *Trichoderma capillare* strains KC2–2 and SLO1–1 were expectedly similar, with introns present in the *cob* and *cox1* coding sequences. The *Trichoderma asperellum* strain TLI also had an intron in *cox1*, but other introns in *cox2* and *cox3*. In contrast, *Trichoderma saturnisporopsis* strain RSI had 20 introns scattered throughout the mitogenome, with a putative sequence for a GIY-YIG endonuclease present in the *cox1* sequence. This resulted in the roughly doubling in the size of the mitochondrial genome for this strain.

### 3.2 Differences in coding sequences of the eight grapevine-isolated strains and 22 other *Trichoderma* mitochondrial genomes

#### 3.2.1 Differences in presence of tRNAs among *Trichoderma* species and strains

**Figure 2:**
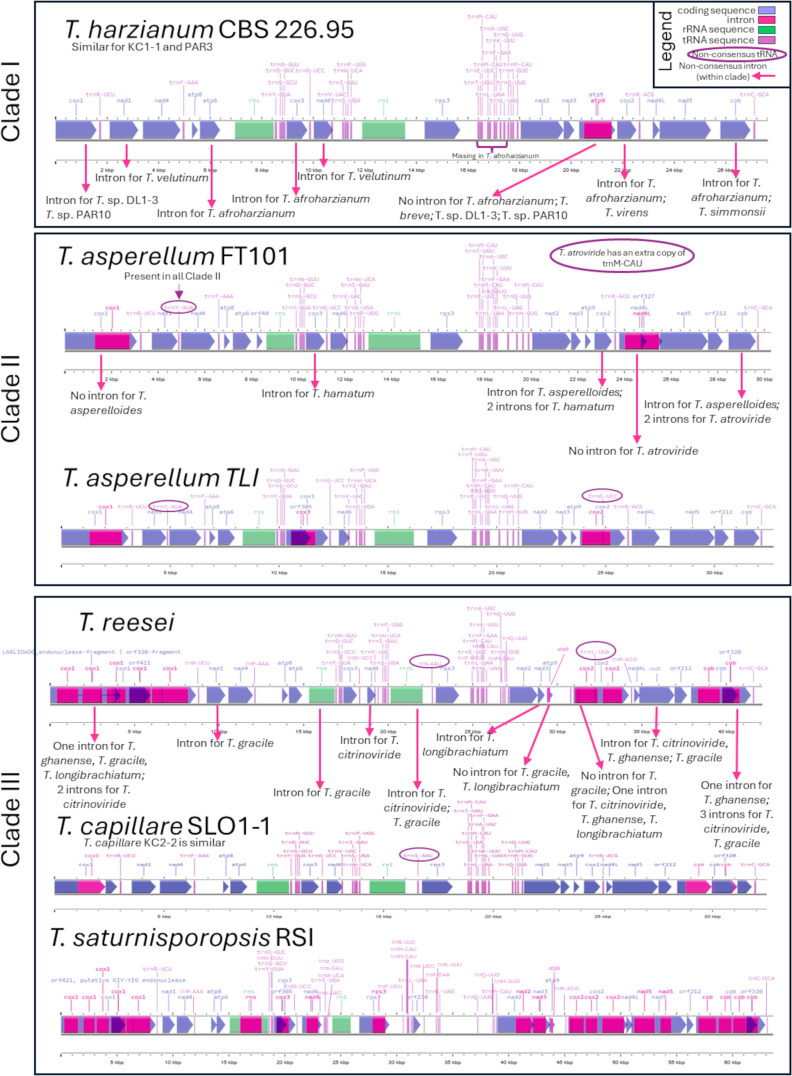
Annotation maps of the different members of the three major clades observed in this study (as identified in **Figure 3**): *Trichoderma harzianum* for clade I; *Trichoderma asperellum* (two strains) for clade II; or *Trichoderma reesei*, *Trichoderma capillare*, and *Trichoderma saturnisporopsis* for clade III. Pink arrows represent introns specific for certain species within a clade. Purple circles represent differences in tRNA among members of the same clade.

All examined *Trichoderma* strains in this study generally had at least the same 25 tRNAs in the mitochondrial genome, one for each amino acid except three copies for methionine (i.e. trnM-CAU), arranged in this order is starting with *cox1* gene: trnR-UCU, trnY-GUA, trnD-GUC, trnS-GCU, trnN-GUU, trnG-UCC, trnV-UAC, trnI-GAU, trnS-UGA, trnW-UCA, trnP-UGG, trnT-UGU, trnE-UUC, trnM-CAU, trnM-CAU, trnL-UAA, trnA-UGC, trnF-GAA, trnK-UUU, trnL-UAG, trnQ-UUG, trnH-GUG, trnM-CAU, trnR-ACG, and trnC-GCA. Differences in the number of tRNA copies for a species or strain from this are noted in **Table 2**, and visualized in **Figure 2**.

**Table 2:** Summary of differences among different *Trichoderma* mitochondrial genomes.

Species	Isolate	tRNA differences	Introns present*
*Trichoderma asperellum*	TLI	Addition of trnF-AAA	*cox1-i1; cox2-i2; cox3-i1; nad4L-i*
*Trichoderma capillare*	KC2-2		*cob-i1; cob-i3; cox1-i1*
*Trichoderma capillare*	SLO1-1		*cob-i1; cob-i3; cox1-i1*
*Trichoderma harzianum*	KC1-1		*atp9-i*
*Trichoderma harzianum*	PAR3		*atp9-i*
*Trichoderma saturnisporopsis*	RSI		*cob-i1; cob-i3; cob-i4; cob-i5; cox1-i1; cox1-i3; cox1-i6; cox1-i7; cox2-i1; cox2-i2; cox2-i3; cox3-i1; nad2-i; nad3-i; nad5-i1; nad5-i2; nad6-i1; rps3-i; rrnS-i*
*Trichoderma* sp. DL1-3	DL1-3		*cob-i3; cox1-i2*
*Trichoderma* sp. PAR10	PAR10		*cox1-i2*
*Trichoderma afroharzianum*	YN065	Only one copy of trnM-CAU; Missing trnA-UGC, trnE-UUC, trnF-GAA, trnK-UUU, trnL-UAA, and trnL-UAG	*atp6-i; cob-i2; cox2-i2; cox3-i2*
*Trichoderma asperelloides*	ZJ116	Addition of trnF-AAA	*cob-i2; cox2-i1; nad4-i*
*Trichoderma asperellum*	FT101	Addition of trnF-AAA	*cox1-i1; nad4L-i*
*Trichoderma atroviride*	ATCC 26799	Extra copy of trnM-CAU	*cob-i1; cob-i2; cox1-i1*
*Trichoderma breve*	T069		
*Trichoderma brevicompactum*	HA032		*cox1-i1; nad6-i2*
*Trichoderma citrinovirde*	IMI 91793		*atp9-i; cob-i1; cob-i2; cob-i3; cox1-i1; cox1-i6; cox1-i7; cox2-i3; nad5-i1; nad6-i1; rrnL-i3*
*Trichoderma gamsii*	KUC1747		
*Trichoderma ghanense*	SC106		*atp9-i; cob-i2; cox1-i1; cox2-i2; nad5-i1*
*Trichoderma gracile*	HK011		*atp6-i; cob-i1; cob-i2; cob-i3; cox1-i1; nad1-i; nad5-i2; rrnL-i2; rrnS-i*
*Trichoderma hamatum*	ThamA		*cox1-i1; cox2-i1; cox2-i2; cox3-i1*
*Trichoderma harzianum*	CBS 226.95		*atp9-i*
*Trichoderma koningiopsis*	POS7		*cox3-i1*
*Trichoderma lixii*	MUT3171		*atp9-i*
*Trichoderma longibrachiatum*	XJ011		*cob-i1; cob-i3; cox1-i5; cox2-i1; nad3-i*
*Trichoderma reesei*	QM 9414		*atp9-i; cob-i1; cob-i3; cox1-i1; cox1-i4; cox1-i5; cox1-i7; cox2-i1; cox2-i2*
*Trichoderma simmonsii*	GH-Sj1		*atp9-i; cob-i2*
*Trichoderma velutinum*	FJ002	Extra copy of trnT-CGU, trnL-AAG, and trnL-CAG; Missing trnL-UAG	*atp9-i; nad1-i; nad6-i1*
*Trichoderma virens*	Gv29-8		*atp9-i; cox2-i2*

* All Trichoderma have rps3 as an intron in the rRNA large subunit gene (*rrnL-i1*).

#### 3.2.2 ATP synthase enzyme subunit and *rps3* genes

A summary of differences of these genes is provided in **Table 2**, **Figure 2**, with the nucleotide positions provided below from the first nucleotide of the particular gene.

For *atp6*, certain species were missing a codon (three nucleotides) at position 7, including *T. capillare* (KC2–2 and SLO1-1), *T. ghanense, T. gracile, T. longibrachiatum*, and *T. reesei*. There also was an intron at position 576 for *T. afroharzianum* and 573 for *T. gracile*, with the intron being 1575bp in length, IA type, and designated here as *atp6*-*i.* This intron also was observed by Wang et al. (2024). For *atp8*, all species had the same length for this gene and no introns were present. For *atp9*, there was a type IA intron at position 182 through 1262 for many of the *Trichoderma* species in the *harzianum* and *reesei* clade: *T. citrinoviride*, *T. ghanense, T. harzianum* (all strains), *T. lixii, T. reesei* (which had an extra 15 nucleotides in this intron)*, T. simmonsii*, and *T. velutinum*. This will be designated here as *atp9-i.* This was similar to findings by Wang et al. (2024), where additional species not analyzed here had this intron as well.

For *rps3*, strain RSI (*T. saturnisporopsis*) had a previously unidentified and unique type IA intron from position 1226 to 2379 of 1154 bp in length, and no introns were observed in the other species examined, designated here as *rps3-i*. It should be noted that the *rps3* gene exists in an intron itself within the rRNA large subunit coding sequence in all *Trichoderma* mitochondrial genomes examined (designated as the intron *rrnL*-*i1*). This was noted by Kwak (2020).

#### 3.2.3 Apocytochrome B (*cob*) and cytochrome c oxidase (*cox*) genes

A summary of differences of these genes is provided in **Table 2**, **Figure 2**, with the nucleotide positions provided below from the first nucleotide of the particular gene.

For *cob*, strain RSI (*T. saturnisporopsis*) had previously unidentified and unique introns present at positions 202 to 1804 (1603 bp length), a type ID intron designated here as *cob-i4*, and 3438 to 4618 (1181 bp in length), a type IB intron designated here as *cob-i5.* The first intron that was not unique to RSI but found in several *Trichoderma* isolates, a type ID intron designated herein as *cob-i*3 was present at consensus position 394 to 1739 (1346 bp in length) in RSI, *T.* sp. DL1-3*, T. capillare* (both strains)*, T. citrinoviride, T. gracile, T. longibrachiatum*, and *T. reesei*. The second intron that was not unique to the RSI strain but found in several *Trichoderma* isolates, a type IB intron designated *cob-i1*, was present at position 1853 to 2912 (1060 in length) in strain RSI, *T. capillare* (both strains)*, T. atroviride*, *T. citrinoviride, T. gracile, T. longibrachiatum*, and *T. reesei*. The last intron in the *cob* gene was type IB, designated *cob-i2*, and began at position 3200 to 5594 (up to 2364 in length) in *T. afroharzianum, T. asperelloides, T. atroviride, T. citrinoviride, T. ghanense, T. gracile*, and *T. simmonsii*. These results were similar to those by Wang et al. (2024).

For *cox1*, most species had a type IB intron, designated herein as *cox1-i1*. This was present in *T. capillare* strain KC2-2, *T. saturnisporopsis* stain RSI, *T. capillare* SLO1-1, *T. asperellum* strain TLI*, T. asperellum, T. atroviride, T. brevicompactum, T. citrinoviride, T. ghanense, T. gracile, T. hamatum*, and *T. reesei* from 1066-2333, 4495-7864, 1066-2333, 1066-2510, 1066-2510, 1066-2344, 1066-2336, 3243-6615, 1066-3170, 1066-4438, 1066-2336, or 4675-8049. Other type IB introns were found in the *cox1* gene, such as an intron designated *cox1-i2* in *T.* sp. DL1-3, and *T.* sp. PAR10 from 219-1464; an intron designated *cox1–3* in *T. saturnisporopsis* stain RSI from 288 to 1546; an intron designated *cox1-i4* in *T. reesei* from 393 to 1669; an intron designated as *cox1-i5* in *T. longibrachiatum* and *T. reesei* from 622–1904 or 1783-3092, respectively; an intron designated as *cox1-i6* in *T. saturnisporopsis* stain RSI and *T. citrinoviride* from 1997–3032 or 738-1776, respectively; and an intron designated *cox1-i7* in *T. saturnisporopsis* stain RSI, *T. citrinoviride*, and *T. reesei* from 3169-4302, 1913-3050, or 3343-4482, respectively.

For *cox2*, *T. saturnisporopsis* stain RSI, *T. asperellum* strain TLI, *T. afroharzianum*, *T. ghanense*, *T. hamatum, T. reesei*, and *T. virens* have a type IB intron, designated *cox2-i2* (from Kwak (2020)), from position 82-1401, 82-1403, 82-1376, 82-1401, 82-1404, 82-1437, and 82-1376, respectively. *T. saturnisporopsis* stain RSI, *T. asperelloides*, *T. hamatum, T. longibrachiatum*, and *T. reesei* have a type IB intron, designated *cox2-i1* (from Kwak (2020)), from positions 1549-2603, 229-1347, 1552-2670, 229-1348, and 1585-2581, respectively; and *T. saturnisporopsis* stain RSI, and *T. citrinoviride* have a type IA intron, designated *cox2-i3*, from positions 3207–4995 or 652-2591. *T. reesei* also had up to 10 smaller insertions from positions 2594 through 2877.

For *cox3*, *T. saturnisporopsis* stain RSI, *T. asperellum* strain TLI, *T. hamatum*, and *T. koningiopsis* had a type IB(3) intron designate as *cox3-i1* from position 220 to 1331, 1337, 1337, or 493, respectively, and *T. afroharzianum* has a type IA intron from position 641 to 2172 designated *cox3-i2*.

#### 3.2.4 NADH dehydrogenase subunit genes

A summary of differences of these genes is provided in **Table 2**, **Figure 2**, with the nucleotide positions provided below from the first nucleotide of the particular gene.

For *nad1*, *T. gracile* and *T. velutinum* have a type IB(3) intron, designated *nad1-i*, from position 637–1684 or 637-1719, respectively. No other introns were observed. For *nad2, T. saturnisporopsis* stain RSI has a type IA intron, designated *nad2-i*, from position 1636-3016. For *nad3*, *T. longibrachiatum* has a type IA intron, designated *nad3-i*, from position 91-1379, and *T. saturnisporopsis* stain RSI has this intron from position 121-1284. For *nad4*, in *T. asperelloides* there was a type IB(3) intron, designated *nad4-i*, from position 1316-1392. For *nad4L*, both analyzed strains of *T. asperellum* had a type IC1 intron, designated *nad4L-i*, from position 240-1716. For *nad5*, *T. citrinoviride, T. ghanense*, and *T. saturnisporopsis* stain RSI have a type ID intron, designated *nad5-i1*, from position 250-2098, 250-2085, or 250-2095, respectively. Also for *nad5, T. saturnisporopsis* stain RSI and *T. gracile* have a type IB3 intron, designated *nad5-i2*, from position 2567–3576 or 718-1722, respectively. For *nad6*, *T. brevicompactum* has a type IA intron, designated *nad6*-*i2*, from position from 247 to 306, and *T. saturnisporopsis* stain RSI, *T, citrinoviride*, and *T. velutinum* had a type IB(3) intron, designated *nad6*-*i1*, from position 626-1676, 626–1681 or 626-1695, respectively.

#### 3.2.5 rRNA coding sequences

A summary of the differences in rRNA coding sequences is provided in **Table 2**, **Figure 2**, with the nucleotide positions provided below from the first nucleotide of the particular gene.

For the rRNA large subunit sequence, all *Trichoderma* had an intron present with the *rps3* gene, called herein as *rrnL*-*i1.* In addition, *T. gracile* has a type IB intron designated *rrnL-i2* from position 331-1679, and *T. citrinoviride* has a type IB intron designated *rrnL-i3* from position 1010-1034.

For the rRNA small subunit sequence, *T. gracile* and *T. saturnisporopsis* had a type II intron designated *rrnS-i* from position 915–2843 or 915-2835, respectively.

### 3.3 Phylogenetic comparisons

**Figure 3:**
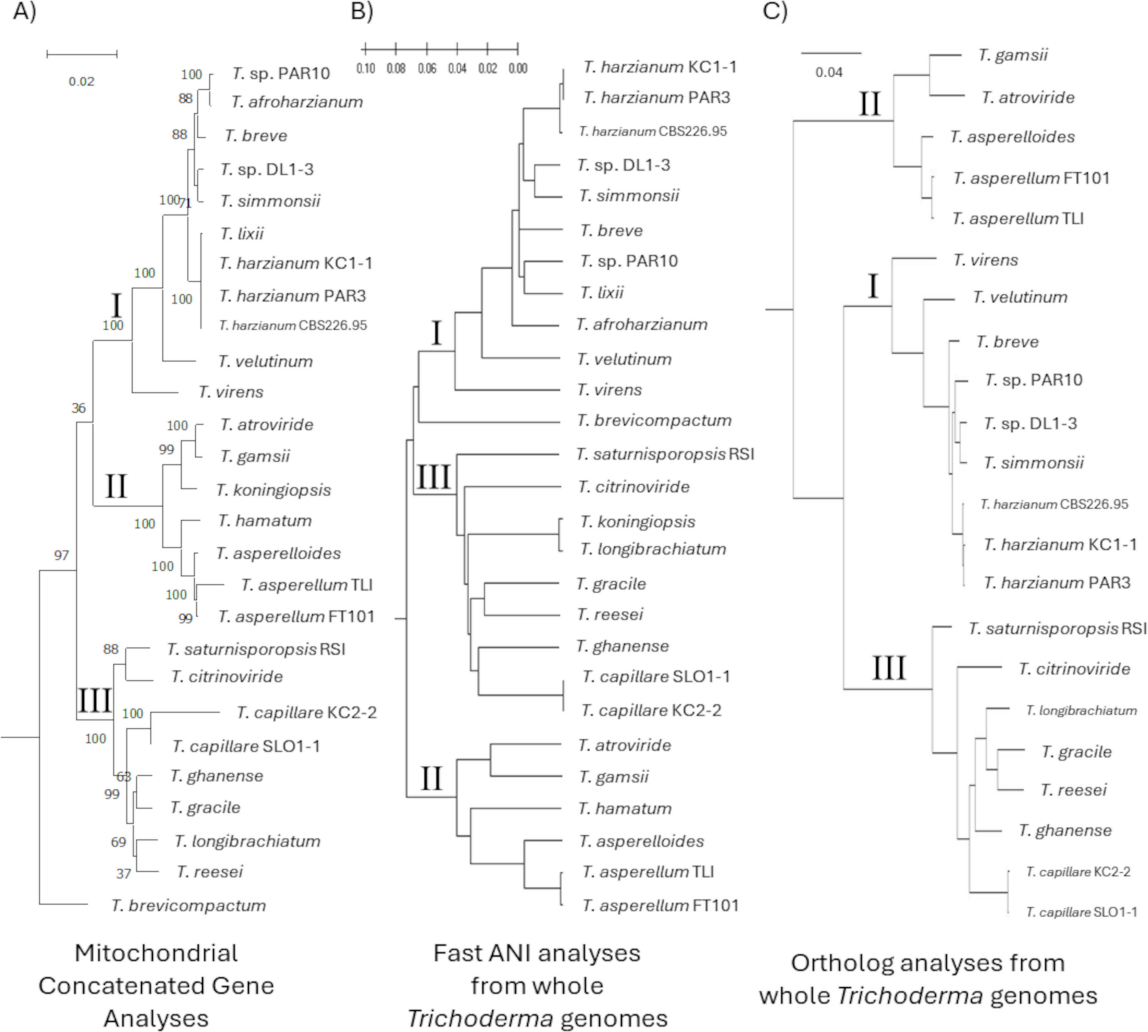
Phylogenetic trees to compare the ability of **(A)** concatenated mitochondrial genomes, **(B)** ANI analyses using whole genome sequences, and **(C)** ortholog analyses using whole genome sequences to resolve species and species relationships. *Trichoderma* clades were consistent: I = *T. harzianum* clade; II = *T. asperellum* clade; or III = *T. reesei* clade. Note that five species (*T. afroharzianum*, *T. brevicompactum*, *T. hamatum*, *T. koningiopsis*, and *T. lixii*) were unable to be included in the ortholog analyses due to lack of protein annotations.

The utilization of mitochondrial genomes to identify and make conclusions about the systematics of *Trichoderma* was made by comparison with techniques using the whole genome, namely the use of average nucleotide identity (ANI) and protein ortholog analyses. This revealed consistency in phylogenetic trees (**Figure 3**), albeit conclusions appeared far more definitive using the whole genome approaches than mitochondria alone. Specifically, it was clear that strains of the same species may have been concluded to be more different in the mitochondrial analyses than using whole genome approaches. Regardless, the three major clades were consistently defined by all three approaches. The greatest differences were observed with the placement with *Trichoderma afroharzianum* and *Trichoderma lixii*.

## 4 Discussion

In this study, the mitochondrial genomes of the species *Trichoderma capillare* (strains KC2–2 and SLO1-1) and *Trichoderma saturnisporopsis* (strain RSI) are reported for the first time, as well as an additional strain of *Trichoderma asperellum* (strain TLI). All of these had the same coding regions and gene order as other *Trichoderma* species. However, the mitochondrial genome of *Trichoderma saturnisporopsis* strain RSI had a particularly large number of introns found present throughout its genome, leading to an almost doubling in the size compared to other species and strains. It remains unknown whether this was limited to this strain or applies to other strains of *Trichoderma saturnisporopsis* as well.

Following the acquisition of the mitochondrial genomes of these four new species, an effort was made to compare these with other *Trichoderma* species and strains to observe the diversity among different mitochondrial sequences. It was observed that tRNA composition was very consistent among different strains, with the major differences being an additional tRNA for phenylalanine in *Trichoderma asperellum* and the related *Trichoderma asperelloides*, additional tRNAs present in *Trichoderma velutinum*, and the absence of eight tRNA sequences in *Trichoderma asperellum.*

The other major differences examined among different mitochondrial genomes was the presence or absence of different introns. *Trichoderma* species in a clade that includes *Trichoderma reesei* (which also includes *Trichoderma capillare, T. citrinoviride*, T*. gracile, T. ghanense, T. longibrachiatum*, and *T. saturnisporopsis*) had the greatest number and diversity of introns. This suggests that these may have had greater activity of LAGLIDADG and GIY-YIG endonucleases affected the mitochondrial genome to potentially result in the accumulation of more introns.

The other consistent trend with an intron was the presence of an intron in the *atp9* sequence, named herein as *atp9-i*, which appears a consistent marker for *Trichoderma harzianum* and closely related species, namely *Trichoderma lixii* and *T. simmonsii.* However, this *Trichoderma reesei* clade also has this intron, and others in the *T. harzianum* lack it (such as *Trichoderma breve*, *Trichoderma* sp. DL1–3 and *T.* sp. PAR10).

Finally, an attempt was made to observe whether mitochondrial genomes could be used to make conclusions about the placement of strains into species and clades, i.e. utility in systematics. This reason is that obtaining mitochondrial genomes can be a comparatively faster process than whole genomes currently, as these have a high copy number and relatively easy to annotate. Likewise, mitochondrial genomes are increasingly available on GenBank and other online resources. Indeed, a greater number of mitochondrial genomes were available than whole genome sequences for the species used in this study, and especially so than genomes with initial protein annotations made. Protein annotations are needed to perform ortholog analyses to make inferences for phylogenetic trees.

Comparing the phylogenetic trees derived from the mitochondrial genomes with those made from using average nucleotide identities (ANI) across the whole genome or by using inferences with orthologs generally revealed consistent clades, with some notable exceptions. For example, the species with Clade II had *T. asperellum* grouped with *T. asperelloides*, and *T. gamsii* with *T. atroviride*. For Clade III, only the placement of *T. capillare* appeared to differ between the mitochondrial genome-derived tree and those based on the whole genome analyses, with *T. capillare* close to *T. ghanense* in the ANI and ortholog trees, but in a separate ancestral branch on the mitochondrial tree. The majority of differences were in Clade I (the *T. harzianum* clade), specially different placements of *T. afroharzianum*, *T. breve, T. lixii*, and *T.* sp. PAR10 in that clade. Furthermore, the placement of *T. brevicompactum* was inconsistent between the phylogenetic methods used.

The difficulty of identifying *Trichoderma* species, especially those closely related to *T. harzianum*, was previously noted by Cai and Druzhinina (2021), who demonstrated that *ITS* regions were unable to resolve species, and even using two preferred alternative housekeeping genes (*rpb2* and *tef1*). The findings herein confirm this may too be the case using a large, but still relatively easy to obtain, sequence in the mitochondrial genome. Indeed, multiple previous studies that used mitochondrial genetic information for phylogenetic placement of species of *Sordariomycetes* fungi, of which *Trichoderma* is a member genus, had varied success (Li et al., 2015; Lin et al., 2015; Deng et al., 2016; Zhang et al., 2017). This said, Wang et al. (2024) demonstrated the usefulness of mitochondrial genomes of different strains of *Trichoderma* in making phylogenic conclusions, as trees generated for mitochondrial genomes in that study were mostly congruent with trees made using nuclear genome phylogeny based on single-copy genes, especially at the main evolutionary branch level. Thus, for putative placements the use of mitochondrial genomes could still be useful and informative (Kwak, 2020), with the acknowledgement that in certain cases such placements should remain putative until whole genome sequence analysis can be completed. Indeed, not all species examined could be used for the ortholog analyses performed herein, as protein annotation was not yet available, and therefore alternative sequence-based identification approaches are desirable until technology and whole genome sequences become readily available and affordable. However, there are issues surrounding the use of mitochondrial genomes in discriminating species that should be acknowledged. Such limitations of using mitochondrial DNA have been previously discussed, including increased recombination events, inconsistent mutation rates, potential heteroplasmy, and other compounding evolutionary processes that affect mitochondrial DNA (Rubinoff and Holland, 2005; Rubinoff et al., 2006). This would affect the ability to form a molecular clock and separate closely related species accordingly (Rubinoff et al., 2006). Despite these limitations, it appears that the congruence of mitochondrial genome-derived phylogenetic trees with those made using nuclear genome-based approaches for the *Trichoderma* genus, as observed in this study and by Wang et al. (2024), suggests that the use of mitochondrial genomes to identify and separate species for this taxon may be quite valid (Xing et al., 2025). Further studies that compare mitochondrial genome-derived trees with those made using nuclear genomes, as well as those based on ecology, morphology, and physiology, are warranted (Samuels and Hebbar, 2016).

Lastly, another limitation of this work in particular was the lack of multiple isolates and strains per species. Thus, acquisition and analysis of additional strains of especially *T. saturnisporopsis*, and the other species as well, is warranted in future research. Additionally, the use of two different sequencing approaches, long-read (for KC2-2) or short-read (for RSI, SLO1-1, and TLI) might have influenced results to some extent, albeit a more in-depth, future study would be needed that use both approaches on all these strains would be needed with a likely hybrid assembly approach to verify accuracy, albeit prior studies have shown consistent results between the two methodologies (Baeza and García-De León, 2022; Slapnik et al., 2024).

In conclusion, mitochondrial genomes could potentially be a useful resource for systematics and the understanding of the evolutionary history of *Trichoderma*, although ultimately analyses of the entire genome are necessary to reach firm conclusions. However, at the time of this writing many *Trichoderma* species do not have whole genomes available. Furthermore, many more strains need to be sequenced to examine natural diversity within a species. The publishing of mitochondrial genomes alongside key genomic sequences (i.e. *ITS, rpb2*, and *tef1*) would be an appropriate place to start until technology allows genomic sequence processing to become readily available and affordable.

## Data Availability

The datasets presented in this study can be found in online repositories. The names of the repository/repositories and accession number(s) can be found in the article/**Supplementary Material**.

## Supplementary material

Supplementary Table 1 Accessions of the whole *Trichoderma* sp. genome sequences used for phylogenetic trees.

Supplementary Figure 1 *Trichoderma* strain mitochondrial genome-containing contig coverage maps for the strains sequenced by Illumina technology (RSI, SLO1-1, and TLI). Note these coverage maps were for untimed and non-circularized contigs.
