# Supplementary material for: Utilizing mitochondrial genome sequences to understand population diversity among Trichoderma species
Source: Front Fungal Biol. 2026 May 15;7:1810157. doi: 10.3389/ffunb.2026.1810157 (PMC13219335; doi:10.3389/ffunb.2026.1810157)
Supplement: Supplementary Table 1 — Accessions of the whole Trichoderma sp. genome sequences used for phylogenetic trees. [file DataSheet1.docx]

Supplementary Material

**Supplementary Table 1.** Accessions of the whole *Trichoderma* sp. genome sequences used for phylogenetic trees.

| Species | Isolate | Accession Number |
| --- | --- | --- |
| *Trichoderma asperellum* | TLI | unpublished, see BioProject PRJNA1101532 |
| *Trichoderma capillare* | KC2-2 | unpublished, see BioProject PRJNA1101532 |
| *Trichoderma capillare* | SLO1-1 | unpublished, see BioProject PRJNA1101532 |
| *Trichoderma harzianum* | KC1-1 | unpublished, see BioProject PRJNA1101532 |
| *Trichoderma harzianum* | PAR3 | JAOPFW010000001 |
| *Trichoderma saturnisporopsis* | RSI | unpublished, see BioProject PRJNA1101532 |
| *Trichoderma* sp. DL1-3 | DL1-3 | unpublished, see BioProject PRJNA1101532 |
| *Trichoderma* sp. PAR10 | PAR10 | unpublished, see BioProject PRJNA1101532 |
| *Trichoderma afroharzianum* | Th6* | JBBLXW010000001 |
| *Trichoderma asperelloides* | T203* | JAJKFY010000111 |
| *Trichoderma asperellum* | FT101 | CP084943 |
| *Trichoderma atroviride* | P1* | CP084935 |
| *Trichoderma breve* | T069 | CM052948 |
| *Trichoderma brevicompactum* | IBT40841* | PXNZ01000001 |
| *Trichoderma citrinoviride* | TUCIM6016* | KZ680207 |
| *Trichoderma gamsii* | T6085* | JPDN02000001 |
| *Trichoderma ghanense* | CCMA-1212* | PPTA01000001 |
| *Trichoderma gracile* | HK011 | JAINEY010000001 |
| *Trichoderma hamatum* | FBL Z32* | JBHOAF010000001 |
| *Trichoderma harzianum* | CBS 226.95 | KZ679675 |
| *Trichoderma koningiopsis* | Z35* | JBHOFB010000001 |
| *Trichoderma lixii* | F014* | JBEBNN020000001 |
| *Trichoderma longibrachiatum* | FL-4* | JAOTPX010000001 |
| *Trichoderma reesei* | QM6a* | GL985056 |
| *Trichoderma simmonsii* | GH-Sj1 | CP075864 |
| *Trichoderma velutinum* | SZMC28001* | JBPBCO010000001 |
| *Trichoderma virens* | Gv29-8 | ABDF02000093 |
| *Fusarium globosum* | NRRL 26131* | JAAQPF010000001 |

**
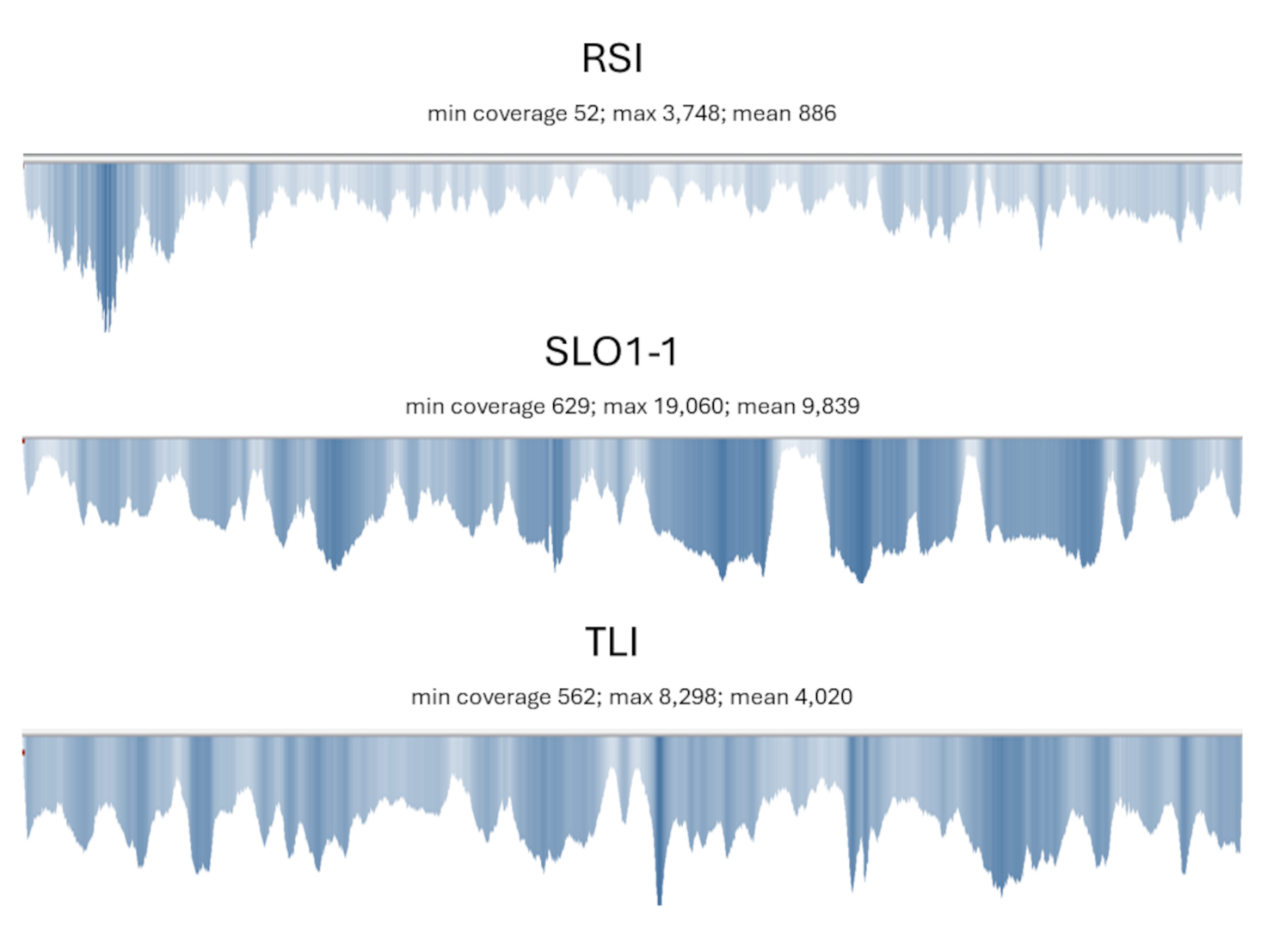
Supplementary Figure 1.** *Trichoderma* strain mitochondrial genome-containing contig coverage maps for the strains sequenced by Illumina technology (RSI, SLO1-1, and TLI). Note these coverage maps were for untimed and non-circularized contigs.
